# Dispersion-Solvent Control of Ionomer Aggregation in a Polymer Electrolyte Membrane Fuel Cell

**DOI:** 10.1038/s41598-018-28779-y

**Published:** 2018-07-16

**Authors:** Ji Hye Lee, Gisu Doo, Sung Hyun Kwon, Sungyu Choi, Hee-Tak Kim, Seung Geol Lee

**Affiliations:** 1 Department of Organic Material Science and Engineering, Pusan National University, 2, Busandaehak-ro 63beon gil, Geumjeong-gu Busan, 46241 Republic of Korea; 2 Department of Chemical and Biomolecular Engineering, Korea Advanced Institute of Science and Technology (KAIST), 291 Daehak-ro, Yuseong-gu Daejeon, 34141 Republic of Korea; 3 Advanced Battery Center, KAIST Institute for the NanoCentury, Korea Advanced Institute of Science and Technology (KAIST), Daejeon, 34141 Republic of Korea

## Abstract

In this study, we examined the influence of the dispersion solvent in three dipropylene-glycol/water (DPG/water) mixtures, with DPG contents of 0, 50, and 100 wt%, on ionomer morphology and distribution, using dynamic light scattering (DLS) and molecular-dynamics (MD) simulation techniques. The DLS results reveal that Nafion-ionomer aggregation increases with decreasing DPG content of the solvent. Increasing the proportion of water in the solvent also led to a gradual decrease in the radius of gyration (R_g_) of the Nafion ionomer due to its strong backbone hydrophobicity. Correspondingly, MD simulations predict Nafion-ionomer solvation energies of −147 ± 9 kcal/mol in water, −216 ± 21 kcal/mol in the DPG/water mixture, and −444 ± 9 kcal/mol in DPG. These results suggest that higher water contents in mixed DPG/water solvents result in increased Nafion-ionomer aggregation and the subsequent deterioration of its uniform dispersion in the solvent. Moreover, radial distribution functions (RDFs) reveal that the (-CF_2_CF_2_-) backbones of the Nafion ionomer are primarily enclosed by DPG molecules, whereas the sulfonate groups (SO_3_^−^) of its side chains mostly interact with water molecules.

## Introduction

Polymer electrolyte membrane fuel cells (PEMFCs) are promising renewable energy sources for automobiles, stationary power generators, and portable devices due to their high energy densities, zero emissions, and fast start-up times, at even low operating temperatures^1–5^. However, there still remain several hurdles that need to be overcome before the large-scale commercialization of PEMFCs for electric vehicles can be realized. Further effort research is required in order to reduce the cost of Pt and Pt-alloy catalysts and to improve their durabilities, while optimizing and enhancing their performance. At the heart of a PEMFC is the membrane electrode assembly (MEA), which includes a polymer electrolyte membrane (PEM), or ionomer membrane, which is sandwiched between two anode- and cathode-catalyst layers. In particular, the catalyst layers (CLs) have very thin three-phase boundaries where the electrochemical reactions take place. Each CL consists of an electron-conductive catalyst phase, an ion-conductive polymer-electrolyte phase, and a porous reactant-transport phase. Consequently, CLs are particularly sensitive and demanding, and further studies into their characteristic morphologies are required in order to improve our understanding of them. Conventional CLs are commonly fabricated from catalyst ink dispersions, which are prepared by mixing a carbon supported platinum catalyst (Pt/C), an ionomer, and the dispersion solvent. The catalyst-layer ink is deposited on a diffusion medium or an electrolyte membrane using a number of deposition methods that include decal transfer, spray coating, die coating, screen printing, and inkjet printing. Molecular interactions between the ionomer and the dispersion solvent control the conformations of the ionomer molecules, which subsequently determine the sizes and distributions of the ionomer aggregates in the dispersion solvent; they also govern CL-ink properties, including viscosity, boiling point, rate of solidification, and ultimately the physical and mass-transport properties of the catalyst layer. Therefore, the selection of an appropriate dispersion solvent, and an understanding of the microstructure of the catalyst ink, is vitally important in order to enhance the final performance of the PEMFC.

Much research has focused on the properties of CL inks and the morphologies of the ionomers in CL inks that contain several dispersion solvents. Pioneering work on the effect of the dispersion solvent on the state of Nafion ionomers in solution was presented by Uchida *et al*.^6,7^ and Shin *et al*.^8^, who found that, depending on the dielectric constant (ε) of the solvent, perfluorosulfonate ionomers (PFSIs) can form solutions (ε > 10), colloids (3 ≤ ε ≤ 10), or precipitates (ε < 3). Fernandez *et al*.^9^ reported the influence of solvent composition and evaporation rate on the microstructure of the catalyst layer and concluded that the dielectric constant of the solvent is a key parameter that needs to be controlled during the preparation of CL inks, which was shown to be directly related to electrode performance. Furthermore, studies on the effect of the dispersion solvent, including methanol, ethanol, 1-propanol, 2-propanol, ethylene glycol, propylene glycol, 1,4-butanediol, glycerol, and their mixtures, among others, on ionomer structure have been conducted using ^19^F nuclear magnetic resonance (^19^F NMR) spectroscopy^10–14^, cryogenic scanning electron microscopy (cryo-SEM)^15^, dynamic light scattering (DLS)^14,16–19^, small angle X-ray scattering (SAXS), small angle neutron scattering (SANS)^13,14,19–38^, and electron spin resonance (ESR) techniques^11,12^, among others^9,39–47^. However, none of these experiments clearly identified ionomer morphologies in CL inks owing to limitation associated with observation scale and the complex inner structures of the CL inks.

Since the design of the ionomer-dispersion solvent is based on the observation that the structures of the Nafion-ionomer aggregates in the CL slurry are highly dependent on the interactions between the dispersion solvent and the ionomers, herein, we conducted a systematic experimental investigation into ionomer microstructure and distribution using the DLS technique and molecular-dynamics (MD) simulations. In this study, we introduce a new ionomer dispersion based on a binary mixture of dipropylene glycol (DPG) and water; this mixture allows the ionomer distribution to be tuned, which affects the power performance of the CL. DPG has a high solvating power for perfluorinated sulfonic acids (PFSAs) and forms nanodispersions of ionomers. Hence, we prepared three types of dispersion solvent, namely water, dipropylene glycol (DPG), and a 1:1 (w/w) DPG/water mixture, and investigated the molecular interactions between the ionomers and the given dispersion solvent as the ionomer-morphology of the solution changed. Upon equilibration of the three types of bulk system, the structural properties of the ionomer in each solvent, which include its radius of gyration (R_g_) and radial distribution function (RDF), as well as the solvation energy of the dispersion solvent, were analyzed from a molecular perspective.

## Experimental Section

### Nafion ionomer aggregation

We used the dynamic light scattering (DLS) technique to quantify the scale of Nafion-ionomer aggregation in each solvent system. Firstly, Nafion powder, which was obtained from a commercial Nafion D520 solution (DuPont, ion-exchange capacity = 1.00 meq/g), was dispersed in DPG (Sigma-Aldrich)/DI-water mixtures (DPG proportions of 100%, 50%, and 0%) by ball milling and subsequent stirring. Size analysis was performed at 25 °C using DLS equipment (Zetasizer nano ZS90, Malvern Co.). In order to ensure that the influence of Nafion on solvent viscosity was negligible, dispersions were diluted to 1 wt% for precise DLS measurements.

### Molecular dynamics simulations

#### Model construction

**Figure 1:**
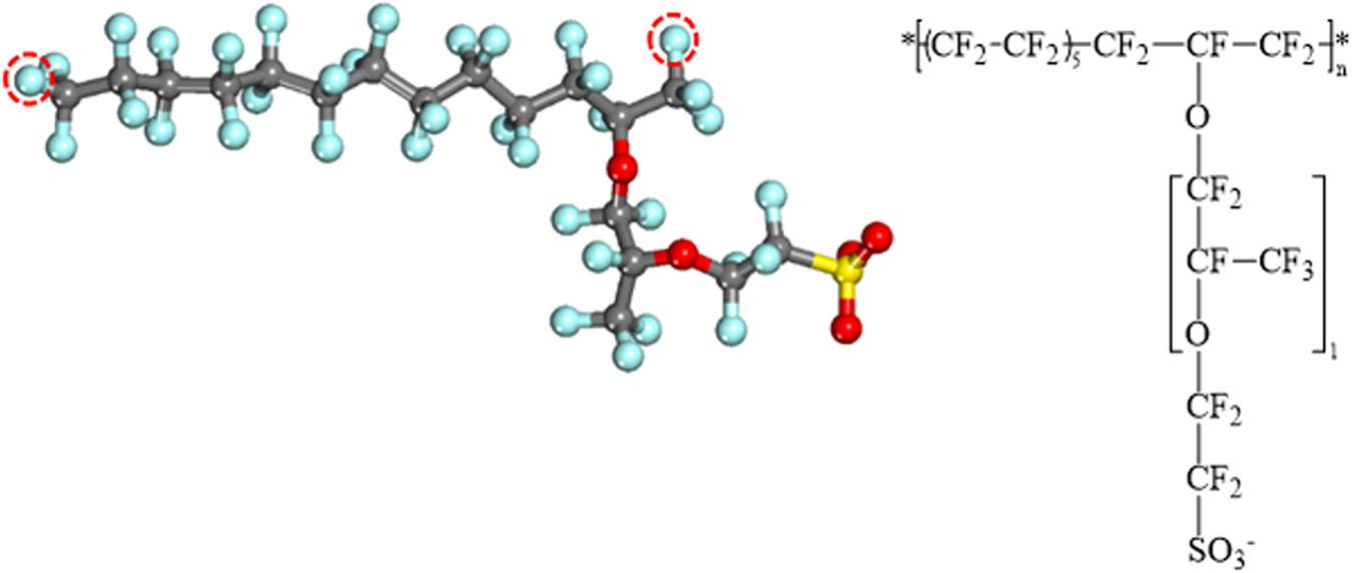
The equilibrated structure (left) and chemical formula (right) of the Nafion monomer. The circles indicate the head and tail atoms in the Nafion-ionomer repeat unit. Carbon, oxygen, fluorine, and sulfur atoms are depicted as gray, red, cyan, and yellow balls, respectively.

The chemical structure of Nafion ionomer with an equivalent weight (EW) of about 1000 g of dry polymer per mol of sulfonic-acid groups was used as the model of ionomer. The chemical formula and structure of the Nafion ionomer employed in this study are illustrated in Fig. 1. The monomeric unit of the Nafion ionomer consists of a backbone of CF_x_ groups and a side chain with two ether linkages terminated by a sulfonic acid group; ten repeat units form the Nafion polymer chain. All sulfonic acid groups in the Nafion ionomer were assumed to be fully ionized (in their sulfonate forms) on the basis of experimental infrared-spectroscopic evidence^48,49^. A number of hydronium ions equal to the total number of sulfonate groups in the Nafion ionomer were added in order to ensure charge neutrality.

Water, DPG, and a 1:1 (w/w) DPG/water mixture were selected as the three solvents for this study. Typically, the composition of a CL ink is controlled on the basis of the weight percentage of each component. The weight ratio of the Nafion ionomer to the dispersion solvent was fixed at 1:4 in this study. We selected the 1:4 ratio of ionomer to solvent in the model due to the limitations on the computational resources and costs. The previous research^50^ reported that the 1:4 (80 wt% solvent content) ratio can be considered as high solvent content to dominate the overall properties of the system by solvents. Thus, the 1:4 ratio of ionomer to solvent may be a good indicator for observing the overall inner-structure of ionomer/solvent system to describe the solvent effects at the atomic scale. Mixtures of the Nafion ionomer and each dispersion solvent were randomly packed into three-dimensional cubic boxes using a Monte Carlo (MC) simulation code. Periodic boundary conditions were applied in all three directions. Simulation details for the three ionomer-dispersed solvent systems are summarized at Table 1.

**Table 1:** MD simulation details.

	Solvent type		
	DPG	DPG/water	water
Simulated cell size (Å)	41.82 ± 0.07	41.36 ± 0.65	42.01 ± 0.06
Number of water molecules	0	1098	2207
Number of DPG molecules	303	149	0

#### Force Field

The DREIDING force field^51^ was employed in this study to describe intermolecular and intramolecular interactions; this force field has been previously used in other fuel-cell studies and various molecular systems^50,52–54^. The F3C^55^ and OPLS-AA^56^ force fields were used for the water and DPG molecules, respectively. The form of the force field is given by:

1 $${E}_{total}={E}_{vdW}+{E}_{electrostatic}+{E}_{bond}+{E}_{angle}+{E}_{torsion}+{E}_{inversion},$$

where E_total_, E_vdW_, E_electrostatic_, E_bond_, E_torsion_, and E_inversion_ are the total energy, and the van der Waals, electrostatic, bond-stretching, angle-bending, torsion, and inversion component energies, respectively. The individual atomic charges were assigned on the basis of quantum-mechanical Mulliken population analyses at the DNP level, using the generalized gradient approximation (GGA) of the Perdew-Burke-Ernzerhof (PBE) functional^57^. The atomic charges on the water molecules were assigned on the basis of the F3C water model. The Particle-Particle Particle-Mesh (PPPM) method^58^ was used to calculate electrostatic interactions.

#### Simulation details

All molecular dynamics (MD) simulations were carried out using the large-scale atomic/molecular massively parallel simulator (LAMMPS) MD code^59^. The equations of motion were integrated using a velocity Verlet algorithm^60^ with a time step of 1.0 fs. A damping relaxation time of 0.1 ps and a dimensionless cell mass factor of 1.0 were employed in the Nose-Hoover temperature thermostat^61,62^ for the canonical (NVT) and isothermal-isobaric (NPT) ensemble MD simulations. All initial models were first relaxed to their local energy minima using the steepest-descent (SD) and conjugate-gradients (CG) optimization algorithms. Following optimization, a 5 ns NVT MD simulation and a subsequent 15 ns NPT MD simulation were performed at 298.15 K in order to fully equilibrate the structure, from which the last 10 ns of each NPT simulation were used for data collection.

## Results and Discussion

### Dynamic light scattering

**Figure 2:**
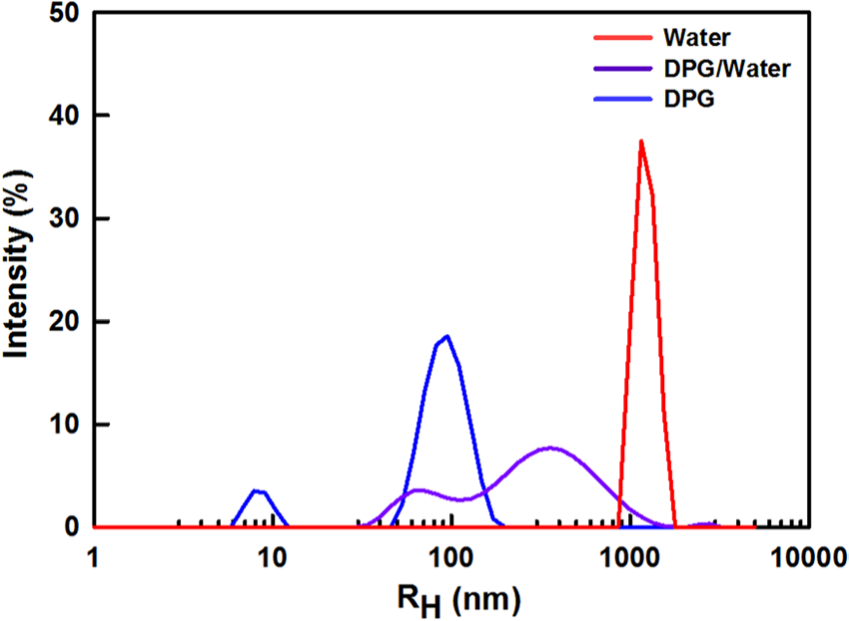
R_H_ intensity distributions for the Nafion-ionomer aggregates in 100% DPG, 50% DPG, and 0% DPG solvents as determined by DLS measurements.

DLS measurements provided a quantitative comparison of the sizes of the ionomer aggregates in the three solvents, namely water (0% DPG), DPG/water (50% DPG), and DPG (100% DPG). The intensity-distribution plot displayed in Fig. 2 clearly reveals that the ionomer-aggregate size strongly depends on the solvent composition. Because the intensity is proportional to the sixth power of the particle diameter, ionomer aggregates that dominate in the catalyst layer in terms of number or volume must be the small particles in the intensity distribution and below analysis was done with the peak at the smaller diameter. The nanoscale hydrodynamic radius (R_H_) of 8 nm determined for 100% DPG reflects the high Nafion-solvating power of DPG. In contrast, pure water (0% DPG) resulted in a micro-scale dispersion (R_H_ ~1 μm) consistent with severe Nafion-ionomer aggregation resulting from poor interactions between water and the hydrophobic Nafion backbone. A submicron-scale Nafion dispersion (R_H_ ~50 nm) was obtained at an intermediate DPG/water composition (50% DPG), due to a combination of interactions between the Nafion ionomer and both the DPG and water. It should be noted that the DLS assumes the aggregates as perfect spheres, which is not true, therefore accurate size or shape of the Nafion ionomer aggregate are not available from DLS data. Nonetheless, it was sufficient to investigate the molecular interactions from the different size scales from tens of nm to micron for the solvents.

### Equilibrated structure

**Figure 3:**
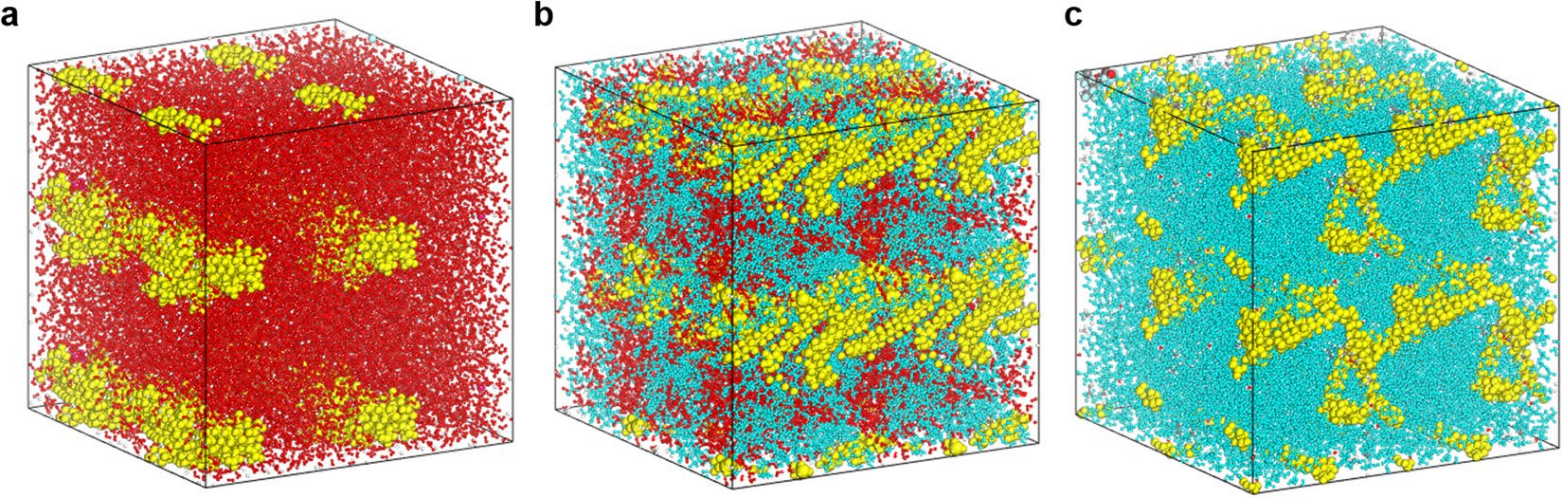
Nafion-ionomer equilibrium structures in 2 × 2 × 2 supercells in (**a**) water, (**b**) 1:1 (w/w) DPG/water, and (**c**) DPG. The Nafion ionomer, DPG, and water molecules are depicted in yellow, cyan, and red, respectively.

**Figure 4:**
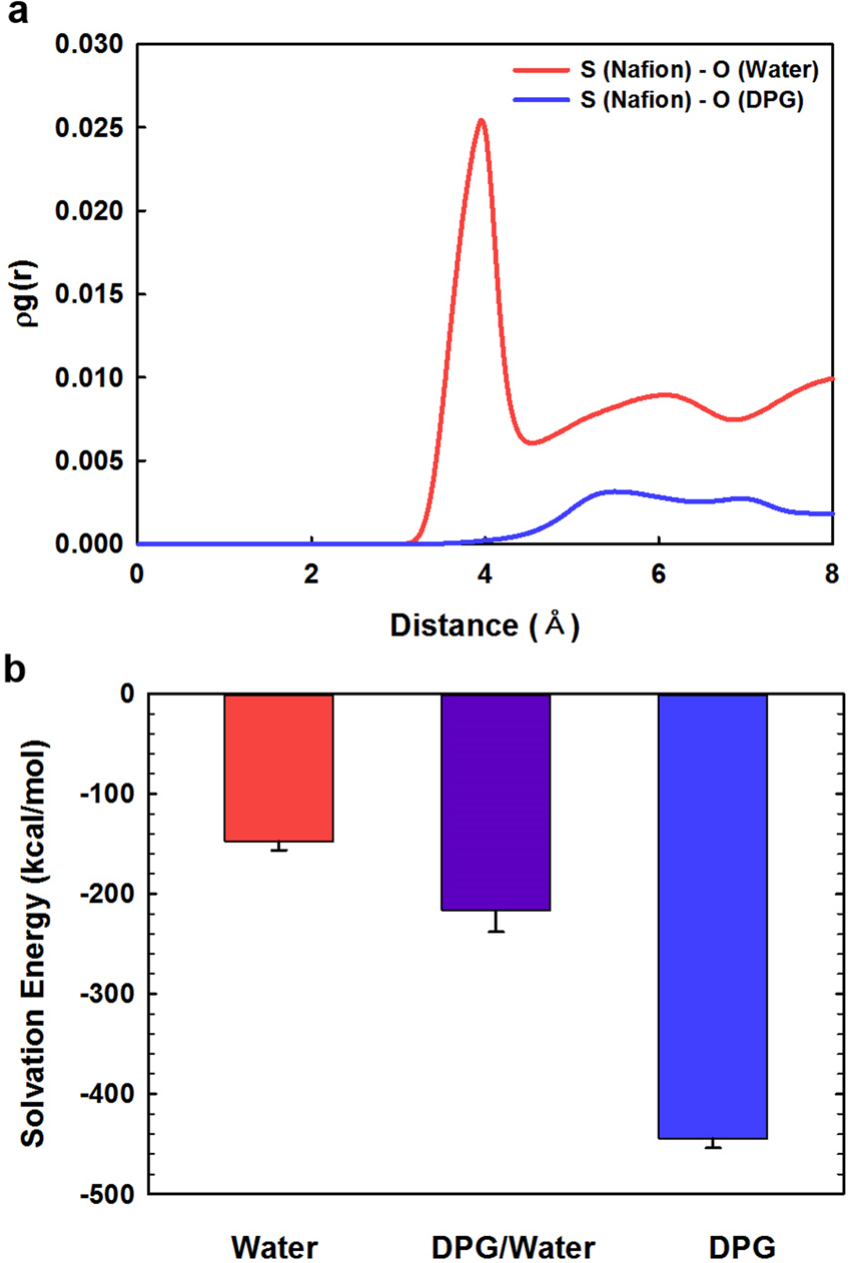
(**a**) RDFs for the Nafion ionomer in the mixed DPG/water dispersion solvent, and (**b**) the solvation energy of the Nafion ionomer in each solvent system.

To explain these experimental observations, a theoretical model was developed in order to acquire detailed atomic-scale Nafion-ionomer-chain information, and to predict the distribution of the Nafion ionomer within the catalyst ink. The dispersion solvent plays a key role in determining ionomer morphology and distribution in this MD study. Final snapshots of the equilibrated structures in the three dispersion solvents, starting from randomly dispersed ionomer structures, are depicted in Fig. 3a–c. As illustrated in Fig. 3, the backbone (-CF_2_CF_2_-) components of the Nafion ionomer tend to aggregate together in water due to the hydrophobic nature of the backbone, which is unwilling to interact with water; the Nafion-ionomer backbone chains form hydrophobic core regions in the aggregated structures. At the same time, the side chains and their sulfonate (SO_3_^−^) groups protrude from this core region into the dispersion-solvent medium, which is ascribable to their high affinities for water molecules because of their hydrophilic and ionic properties. On the contrary, the majority of the (-CF_2_CF_2_-) backbones of the Nafion appear to be extended and remain in a dispersed (or non-localized) state in DPG, which is due to favorable hydrophobic interactions between the backbones of the Nafion ionomer and the DPG solvent. Furthermore, the relatively strong hydrophobicity of the DPG molecule, compared to water, results in the localization of the hydrophilic sulfonic sulfonate groups (SO_3_^−^) of the Nafion ionomer. The equilibrated structure of the Nafion ionomer did not appear to adopt any special backbone or side-chain aggregation in the mixed DPG/water solvent. Detailed analysis of the Nafion ionomer microstructure in the DPG/water mixture was facilitated by radial distribution function (RDF) calculations. The RDF (*g*_*A−B*_*(r)*) is a density function that describes the probability of finding atoms A and B at a distance *r* averaged over the equilibrium trajectory. This function reflects the characteristics of the microstructure; it can be used to reveal the essence of the interactions that occur between non-bonding atoms and can be applied to the structural investigations of solids and liquids. The RDF is calculated by:

2 $${g}_{A-B}(r)=(\frac{{n}_{B}}{4\pi {r}^{2}dr})/(\frac{{N}_{B}}{V}),$$

where *n*_*B*_ is the number of *B* particles located at a distance *r* in a shell of thickness *dr* from particle *A*, *N*_*B*_ is the number of *B* particles in the system, and *V* is the total volume of the system. Using this function, it is possible to determine the environment that the guest molecules are in. In order to directly compare intensities, the products of the pair correlations and number densities (*ρg*_*A−B*_ *(r)*) are used instead of *g*_*A−B*_*(r)*. Figure 4a displays the RDFs for interactions between the Nafion ionomer and the DPG and water molecules in the mixed DPG/water dispersion solvent. Interestingly, the equilibrated structure of the Nafion ionomer in the DPG/water mixture appears to have the Nafion ionomers mainly surrounded by DPG molecules, as shown in Fig. 4a. In contrast, the water molecules appear to be located on the outsides of the DPG molecules that cover the Nafion ionomers. Figure 4a clearly shows that the first S(Nafion)-O(water) pair peak occurs at a noticeably shorter distance and is more intense than the analogous S(Nafion)-O(DPG) pair peak. This indicates that the regions in the vicinity of the sulfonate groups of the Nafion-ionomer side chain are more populated by water molecules than DPG molecules, which is ascribable to strong electrostatic and hydrophilic interactions between the ionic sulfonate groups of the Nafion ionomer and the polar water molecules. Consequently, the RDF analysis is consistent with the intuitional equilibrium structure.

### Radius of gyration

We also calculated ionomer radii of gyration in order to quantitatively analyze the form of the ionomer in the dispersion solvent. The radius of gyration (R_g_) of an ionomer is defined as the average-squared distance of any point in the object from its center of mass, according to:

3 $${R}_{g}={(\frac{1}{N}{\sum }_{i=1}^{N}{({R}_{i}-{R}_{cm})}^{2})}^{1/2},\,$$

where *R*_*i*_ denotes the position vector of the *i*th atom in the ionomer chain, *R*_*cm*_ denotes the position vector of the center of mass of the ionomer chain, and *N* is the total number of atoms in the ionomer chain at a given time. The R_g_s of Nafion-ionomer chains depend largely on the type of dispersion solvent. The R_g_ of the Nafion ionomer is shorter with increasing weight-fraction of water in the solvent, through aggregation. The average R_g_ values of the Nafion ionomer in each solvent were determined to be 13.6 ± 0.4 Å in water, 14.7 ± 0.7 Å in 1:1 (w/w) DPG/water, and 17.2 ± 1.0 Å in DPG. These trends are consistent with the visually observable trends depicted in Fig. 3a–c. Obviously R_H_ and R_g_ are not the same values to compare the results, however, at the atomic scale, R_g_ also gives us the valuable information to describe the interaction between the ionomer and the selected solvents. Accordingly, the ionomer chain in water has smaller R_g_ than DPG, because the hydrophobic main chain (-CF_2_CF_2_-) of ionomer tends to aggregate together in water due to the hydrophobic nature of the backbone. Meanwhile, the ionomer relatively well interacts with DPG solvents that leads higher R_g_ value than that of in water.

### Solvation energy

Few previously reported studies have investigated the importance of molecular interactions between ionomers and the dispersion solvent. Kim *et al*.^38^ reported that the ability of the solvent to mobilize the Nafion ionomer significantly influences the CL structure. They found that CLs fabricated using solvents with high main-chain mobilities created higher levels of intimate contact at triple-phase boundaries, which were mainly due to strong interactions between solvent molecules and the Nafion ionomers; this, in turn enhances electrochemical performance. Furthermore, the Los Alamos National Laboratory group^13,36,38^ investigated the morphologies of Nafion in various solvents by SANS and examined the corresponding electrochemical and mechanical properties. They suggested that fuel-cell-performance durability can be controlled by tuning the properties of the interface between the catalyst and the ionomer through the judicious choice of dispersion medium. Hence, in order to better quantify the interactions between the Nafion ionomer and the dispersion solvent, the solvation energies of the ionomer in the three dispersion solvents were calculated. The solvation energy (*ΔE*_*solvation*_) is used to measure the relative solubility of a polymer in a solvent and is given by:

4 $${\rm{\Delta }}{E}_{solvation}={E}_{system}-({E}_{ionomer}+{E}_{solvent}),$$

where *E*_*system*_ is the total energy of the ionomer in the solvent, *E*_*ionomer*_ is the energy of ionomer, and *E*_*solvent*_ is the energy of the solvent. A negative solvation energy indicates good dispersion or solvation of the Nafion ionomer. As shown in Fig. 4b, the solvation energies of the Nafion ionomer are −147 ± 9, −216 ± 21, and −444 ± 9 kcal/mol in water, 1:1 (w/w) water/DPG, and DPG, respectively. The solvation energy of the Nafion ionomer in DPG is the most negative among the three solvents, which indicates that the Nafion ionomer is well dispersed in DPG. Nafion-ionomer dispersion worsens with increasing levels of water in the solvent, which is in good agreement with the DLS data and the MD results previously discussed.

## Conclusions

Herein, we developed a molecular-level understanding of the structure and dynamics of Nafion ionomers in three types of dispersion solvent using DLS and MD techniques. We used three dispersion solvents with different percentages of DPG in water, namely 100, 50, and 0% (w/w). From an experimental perspective, the ionomer-aggregate size increased in the following order: DPG (R_H_ ~8 nm) <DPG/water (R_H_ ~50 nm) <water (R_H_ ~1 μm). Severe Nafion-ionomer aggregation was observed with increasing levels of water in the solvent. Combining the radius-of-gyration (R_g_) values of the Nafion ionomers with visual inspections of the equilibrated configurations clearly revealed that increasing water content in the mixed DPG/water solvents resulted in reduced ionomer R_g_ values that follow the order: water (13.6 ± 0.4 Å) <water/DPG (14.7 ± 0.7 Å) <DPG (17.2 ± 1.0 Å). Likewise, Nafion-ionomer solvation-energy calculations predict that solvation energy increases with increasing water content, and follows the order: (−444 ± 9 kcal/mol) <DPG/water (−216 ± 21 kcal/mol) <water (−147 ± 9 kcal/mol). Among the three solvents, the Nafion ionomer dispersed in DPG exhibited the most negative solvation energy, which indicates that the ionomer retains a well-dispersed state in the DPG system. Meanwhile, Nafion-ionomer aggregation worsened in water. Finally, RDF analyses revealed that the (-CF_2_CF_2_-) backbones in the Nafion ionomers are predominantly surrounded by DPG molecules, which is ascribable to hydrophobic interactions, whereas the side-chain sulfonate groups (SO_3_^−^) strongly interact with water molecules due to electrostatic interactions.
